# Efficacy and safety of atropine to control myopia progression: a systematic review and meta-analysis

**DOI:** 10.1186/s12886-020-01746-w

**Published:** 2020-12-07

**Authors:** Congling Zhao, Chunyan Cai, Qiang Ding, Hongbin Dai

**Affiliations:** 1 Aier Eye Hospital of Wuhan university, Wuhan, Hubei Province China; 2 Department of Gastroenterology, Tongji Hospital, Tongji Medical College, Huazhong University of Science and Technology, Wuhan, Hubei Province China

**Keywords:** Atropine, Myopia progression, Meta-analysis

## Abstract

**Background:**

The effect and safety of atropine on delaying the progression of myopia has been extensively studied, but its optimal dose is still unclear. Therefore, the purpose of this meta-analysis is to systematically evaluate the safety and effectiveness of atropine in controlling the progression of myopia, and to explore the relationship between the dose of atropine and the effectiveness of controlling the progression of myopia.

**Methods:**

This work was done through the data searched from PubMed, MEDLINE, EMBASE, and the Cochrane Central Register of Controlled Trials. The Cochrane Handbook was also used to evaluate the quality of the included studies. In addition, a meta-analysis was performed using Revman5.3 software.

**Results:**

A total of 10 randomized controlled trials (RCTs) were included. Myopia progression was mitigated greater in the atropine treatment group than that in the control group, with MD = − 0.80, 95% CI (− 0.94, − 0.66) during the whole observation period. There was a statistical difference among 0.05, 0.5, and 1.0% atropine (*P* = 0.004). In addition, less axial elongation was shown, with MD = − 0.26, 95% CI (− 0.33, − 0.18) during the whole observation period.

**Conclusion:**

The effectiveness of atropine in controlling the progression of myopia was dose related. A 0.05% atropine was likely to be the optimal dose.

## Background

Myopia is a multifactorial disease caused by the uncoordinated development of various parts of the eyeball during the process of emmetropization, which is affected by the environment and genes. It is a mismatch between the optical power and length of the eye, causing the incoming light to be focused in front of the retina. It was the most common eye disease in children and adolescents, and has grown rapidly worldwide over the past few decades, especially in East Asian regions where the prevalence of myopia in young adults was around 80–90% [1]. It has been predicted that 4.8 billion people in the world would be myopic by the year 2050, which means that 50% of children would become myopic 30 years later [2].

Myopia have an impact on children’s academic performance, children’s physical activity, psychological development and employment choices. Children with an early onset of myopia, always accompanied with high progression rates, had a higher incidence of high myopia, and a greater risk of glaucoma, cataract, myopic maculopathy, retinal detachment and choroidal neovascularization [3]. Myopia, an urgent public health issue, is the leading cause of preventable blindness in children and adolescents [4].

Currently, there are several approaches to slow down the progression of myopia. First, an increased outdoor activities, and a reduced near work or study could delay the progress of myopia [5], but the outdoor time was limited because of the high educational pressure. Second, compared to emmetropic and hyperopic counterparts who demonstrated relative peripheral myopia, people with myopia displayed relative peripheral hyperopia. Orthokeratology lens could shift the relative peripheral refraction in the myopic direction [6], slow the axial elongation and thus help to delay the myopia progression [7]. However, orthokeratology lens is not appropriate for all the patients, such as patients with severe dry eye or keratitis. Third, atropine, an anticholinergic blocking agent, plays an important role in the different kinds of ocular tissues and slowed the axial elongation of the eye and the myopia progression [8].

Although many studies have demonstrated the effectiveness of atropine in controlling myopia, its optimal dose is still under research and has not been approved by the FDA [9]. Therefore, a meta-analysis was conducted in this work to systematically evaluate the safety and effectiveness of atropine in controlling the progression of myopia, and to explore the relationship between the dose of atropine and the effectiveness of controlling the progression of myopia.

## Methods

This meta-analysis of prospective randomized controlled trials (RCTs) was performed according to the PRISMA statement. The PRISMA Checklist was shown in the Supplementary Dataset. No protocol was used for this meta-analysis.

### Information source and search strategy

A purposive literature search was conducted in PubMed, MEDLINE, EMBASE, and the Cochrane Central Register of Controlled Trials using Medical Subject Headings (MeSH) and free words closely related with myopia and atropine. ((((((((((((Atropine)) OR (Atropinol)) OR (Atropine Sulfate)) OR (Sulfate, Atropine)) OR (Atropine Sulfate Anhydrous)) OR (Anhydrous, Atropine Sulfate)) OR (Sulfate Anhydrous, Atropine)) OR (AtroPen)) OR (Atropin Augenöl)) OR (Augenöl, Atropin))) AND (((((Nearsightednesses)) OR (Nearsightedness)) OR (Myopias)) OR (Myopia)) was used for searching the PubMed. We also searched clinicaltrials.gov and the reference lists of published reviews to find additional relevant studies. The final search date was January 20, 2020. It is noted that only studies published in English were used.

### Eligibility criteria

The included studies must meet the following criteria:

1. A randomized placebo-controlled clinical trials.
2. Spherical equivalent refraction more than − 0.25D measured by cycloplegic autorefraction was diagnosed with myopia.
3. All patients were under 18 years old.
4. Atropine was used for at least 1 year.
5. The study reported at least the annual rate of myopia progression.

Congling Zhao and Chunyan Cai independently reviewed title, abstract, and full-length article to identify potentially eligible articles using the criteria listed above. Disagreements regarding eligibility were resolved through a discussion with Qiang Ding. When a study was reported more than once, only the latest study was included to avoid double inclusion of data. When a study contained different doses of atropine, only the dose recommended by the study was included. The list of exclusion studies and reasons for exclusion were shown in the Supplementary Dataset.

### Data extraction

Two reviewers (Congling Zhao and Qiang Ding) independently extracted data using the pre-established extraction tables, including the following: (1) Basic characteristics of the study, including the name of the first author, year of publication, and follow-up time (2) Basic characteristics of the patients, including the age of the patients, equivalent spherical power before treatment, changes of cycloplegic spherical equivalent, changes of axial elongation, adverse reactions, etc.

### Qualitative assessment

The quality of the included studies was assessed by the Cochrane Handbook, including 6 items: random sequence generation, allocation concealment, blinding of participants and personnel, blinding of outcome assessment, incomplete outcome data, selective reporting, and other biases. Two reviewers determined the risk of bias which had three options (low, high, and unclear). When necessary, we contacted the authors of the studies to obtain the full text or related information for an accurate assessment.

### Statistical analysis

Review Manager (version 5.3; Cochrane Collaboration) was used for data analysis. The statistical heterogeneity of included studies was tested by the Cochrane I^2^ test. If I^2^ was 50% or less, indicating a low-to-moderate heterogeneity, a fixed-effect model was used. If I^2^ was higher than 50%, indicating a high degree of heterogeneity, a random effects model was applied. MD with a 95% confidence interval (CI) was used to estimate the effectiveness. A sensitivity analysis was performed by excluding the included studies one by one.

## Results

### Search results

**Fig. 1:**
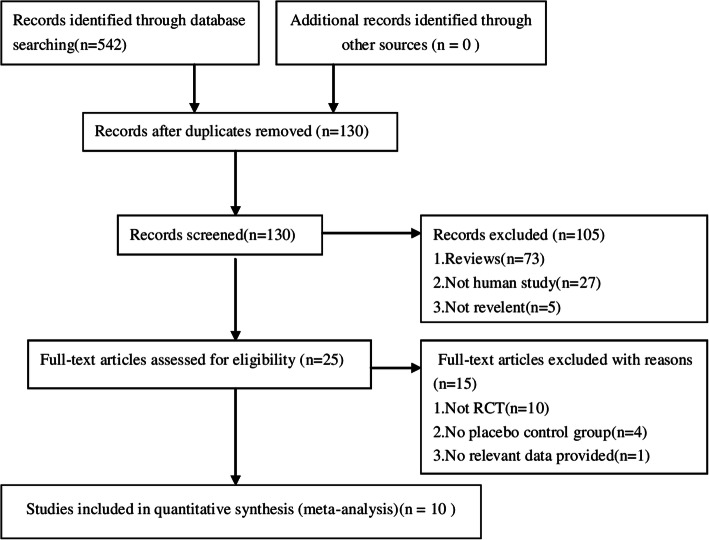
PRISMA Flow Diagram of the Literature Search Process

A total of 542 studies were retrieved. Finally, 10 studies were included in this meta-analysis. The basic characteristics of the 10 studies are shown in the Table 1. There were 809 patients in the atropine group and 814 patients in the control group. 0.05% atropine was used in one study, 0.5% atropine was used in five studies, and 1.0% atropine was used in the other four studies. The literature screening process is shown in Fig. 1.

**Table 1:** Basic characteristics of included studies

Source	Country/Area	Follow- up, M	Included Atropine Dose, %	Age, Year	Baseline Refraction, Diopter (Mean ± SD)	Experimental group	Control group	Total No. of Patients (test group/control group)
Chua et al., 2005 [10]	Singapore	12	1	6–12	−3.58 ± 1.17	1% Atropine	placebo	156/190
Hsiao et al., 2005 [11]	Taiwan	18	0.5	<18	−3.26 ± 0.15	0.5%Atropine+Multi-focal	Multi-focal lenses	66/61
Kumaran et al., 2015 [12]	Singapore	36	1	6–12	−3.36	1% Atropine	placebo	147/166
Polling et al., 2016 [13]	Europeans	12	0.5	<18	−6.6 ± 3.3	0.5% Atropine	placebo	60/17
Shih et al., 2001 [14]	Taiwan	18	0.5	6–13	−3.28 ± 0.13	0.5%Atropine+multi-focal	multi-focal glasses	66/61
Shin et al., 1999 [15]	Taiwan	12	0.5	6–13	−4.89 ± 2.06	0.5,0.25,0.1% Atropine	placebo	41/49
Wang et al., 2017 [16]	China	12	0.5	5–10	−1.3 ± 0.4	0.5% Atropine	placebo	63/63
Yam et al., 2018 [17]	China	12	0.05	4–12	−3.98 ± 1.69	0.05,0.025,0.01% Atropine	placebo	110/111
YEN et al., 1989 [18]	Taiwan	12	1	6–14	−1.523 ± 0.960	1% Atropine	placebo	32/32
Yi et al., 2015 [19]	China	12	1	7–12	−1.23 ± 0.32	1% Atropine	placebo	68/64

### Methodological quality evaluation

**Fig. 2:**
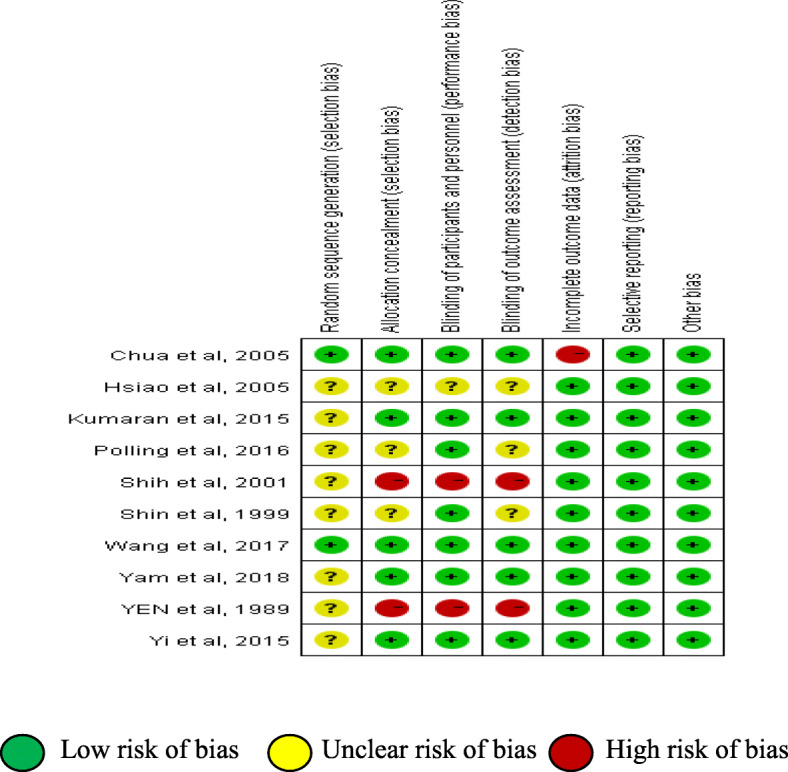
The Results of the Methodological Evaluation. Low risk of bias. . Unclear risk of bias.  High risk of bias

The results of the methodological evaluation according to the Cochran Handbook are shown in Fig. 2. Only two studies reported the generation of random sequences, of which one study was conducted [10] through a computer-generated randomization list and the other [5] through a computer SAS package.

### Efficacy analysis

#### Spherical equivalent refraction

**Fig. 3:**
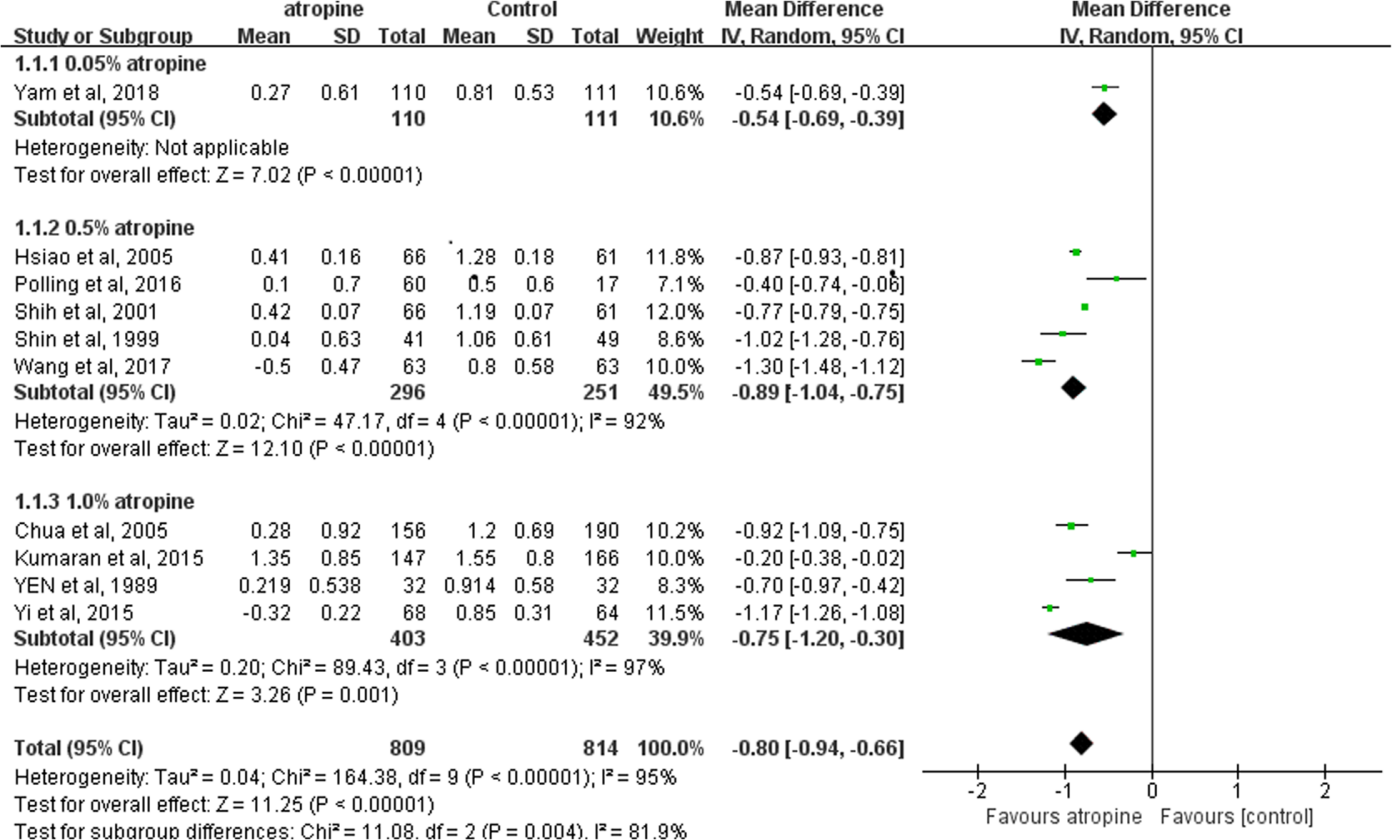
Forest Plots of the Effect of Atropine on Refraction. The progression of myopia was defined as the change in spherical equivalent refractive error relative to the end point. For this scale, negative value indicated myopia improvement and positive value indicated myopia progression. SD, standard deviation. CI, confidence interval

The ten studies all reported the changes of equivalent spherical power. The overall heterogeneity I^2^ was 95%, so a subgroup analysis was performed using a random effects model. The less myopia progression was shown in 0.05% atropine group (MD, − 0.54; 95% CI, − 0.69 to − 0.39; *p*<0.05), 0.5% atropine group (MD, − 0.89; 95% CI, − 1.04 to − 0.75; *p*<0.05), 1% atropine group (MD, − 0.75; 95% CI, − 1.20 to − 0.30; *p*<0.05) than that of the control group during the whole observation period. The overall MD was − 0.80 (95% CI − 0.94 to − 0.66). There were statistical differences between the atropine group and the control group (*P* = 0.004) (See Fig. 3).

#### Axial length

**Fig. 4:**
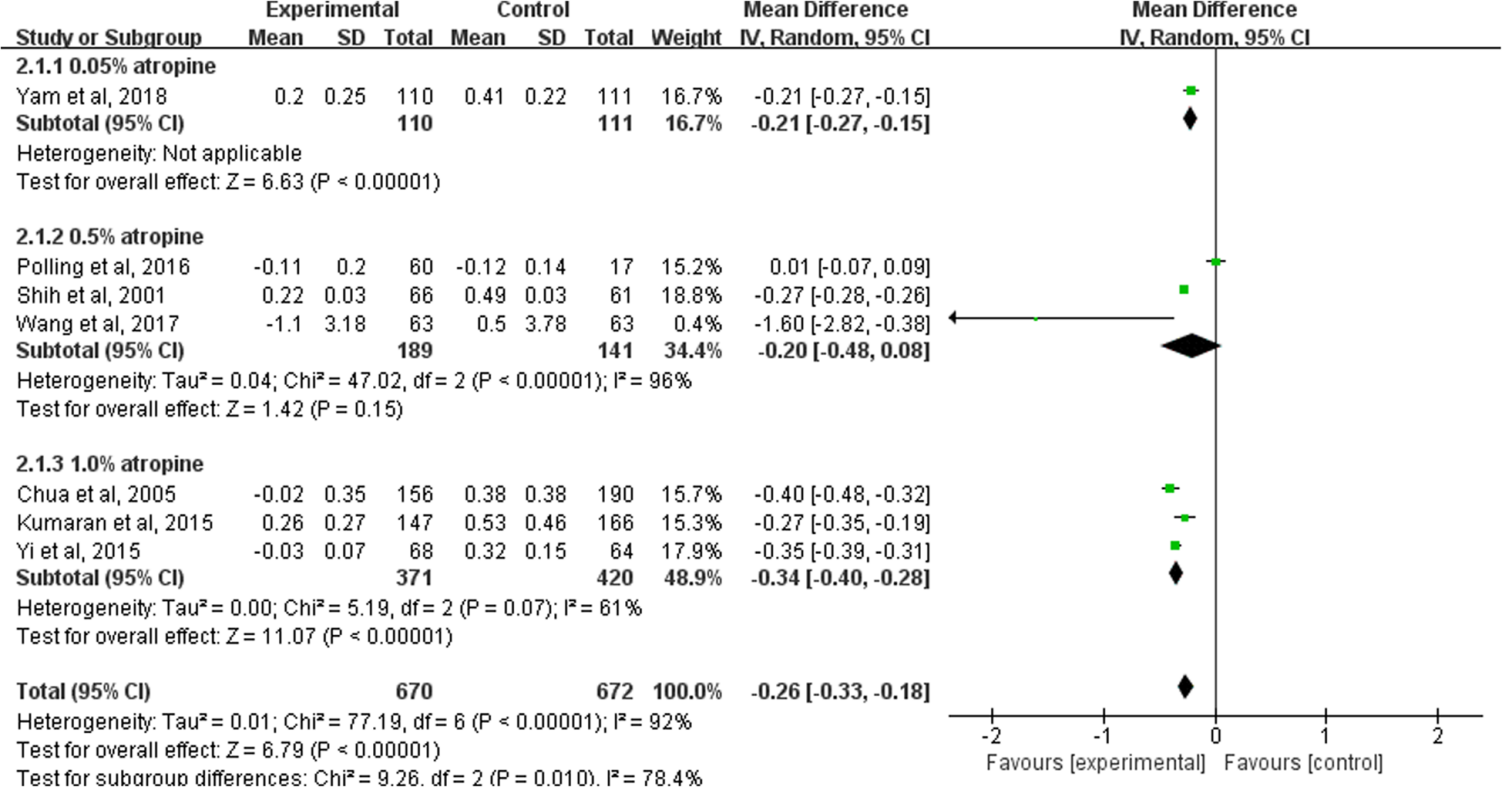
Forest Plot of the Effect of Atropine on Axial Length. Changes in axial length was defined as end point value subtracted by baseline value. For this scale, negative value indicated myopia improvement and positive value indicated myopia progression

Seven studies reported the changes for the axis of the eyes. The date showed a less axial elongation in 0.05% atropine group (MD, − 0.21; 95% CI − 0.27 to − 0.15), 0.5% group (MD, − 0.20; 95% CI − 0.48 to 0.08) and 1% atropine group (MD, − 0.34; 95% CI − 0.40 to − 0.28) than that of the control group during the whole observation period. The overall MD was − 0.26 (95% CI − 0.33 to − 0.18; *P*<0.05) (See Fig. 4).

#### Adverse effects

A total of five studies showed the adverse effects (Table 2). Among them, the most common adverse effect was photophobia, and the others included allergy, headache, blushing, and gastrointestinal reaction. No serious complications were found at any dose of atropine.

**Table 2:** The adverse effects on the different dose of atropine studies

Source	Atropine Dose, %	Adverse effects
Chua et al.,2005 [10]	1	No serious adverse events. Reasons for withdrawal: allergic or hypersensitivity reactions or discomfort (4.5%), glare (1.5%), blurred near vision (1%), logistical difficulties (3.5%), and others (0.5%).
Polling et al., 2016 [13]	0.5	Photophobia (72.4%); reading problems (37.7%); headaches(22.4%); systemic flushes (only in a minority); pain in the eye, irritated eyes, overflow of tears, trouble with depth perception, cosmetically disfiguring pupils, and an unpleasant taste in mouth (all reported only in one patient).
Yam et al.,2018 [17]	0.05	Gastroenteritis, influenza, or asthmatic attack(1 case)
YEN et al., 1989 [18]	1	Photophobia (100%), No systemic or ocular complications
Yi et et al., 2015 [19]	1	No complain

### Sensitivity analysis and publication bias

We performed a sensitivity analysis for the spherical equivalent refraction and the changes for the axis of the eyes. When Wang’s study [16] was excluded individually from this study, the random-effect pooled estimate for the subgroup differences was least significant (*P* = 0.02,I^2^ = 75.1%). The myopia progression was − 0.79D, 95% CI: (− 0.89, − 0.61), similar to that of all the included studies. Therefore, the original result was robust. There were no significant differences between the atropine group and the control group. Funnel plots did not suggest significant publication bias (See Supplementary Dataset).

## Discussion

Myopia is a widespread eye disease in the world. Every year, myopia-related complications causes a huge socioeconomic burden and especially progressive high myopia even may lead to potentially blinding complications. Currently, the best treatment strategy is to control the progression of myopia.

In this meta-analysis, less myopia progression was shown in the atropine treatment group than that of the control group during the whole observation period, with MD = − 0.80, 95% CI (− 0.94, − 0.66). Moreover, there was a statistical difference among 0.05, 0.5, and 1.0% atropine groups (*P* = 0.004). Less axial elongation was also shown in the atropine treatment group, with MD = − 0.26, 95% CI (− 0.33, − 0.18). This confirmed the role of atropine in myopia and suggested that its effectiveness was related to its dose [20], and 0.05% atropine could effectively control the progression of myopia [21].

Song et al. identified that the effectiveness of atropine was related to its dose. A low dose of atropine worsened the progression of myopia. 0.5 and 1.0% of atropine could safely and effectively control the progression of low to moderate myopia [22]. However, the meta-analysis done by Song et al. only included 6 studies in 2011. In addition, the low-dose atropine only included 0.1 and 0.25% and no placebo control was used. Therefore, it is impossible to determine whether the low-dose atropine is ineffective or if it has a worse effectiveness than the higher dose of atropine.

Gong et al. reported that the effectiveness of atropine was independent of its dose, but its side effects were dose-dependent [23]. However, it included the Cohort study which had insufficient evidence.

A recent 2-year follow-up observation [24] in children in the United States found that 0.01% atropine could effectively control the progression of myopia. A meta-analysis [25] published last year verified the effectiveness of 0.01% atropine on myopia, but it did not show the effectiveness of other doses.

When atropine was discontinued after 1 year usage in the atropine group, myopia progressed faster than that of the placebo group [26], especially for a high-dose atropine (low-dose atropine cases rebounded less after discontinuation) [27]. Therefore, the effectiveness of rebound was closely related to its dose.

This meta-analysis provides evidence-based medical evidence for the use of atropine in controlling the progression of myopia by including only high-quality RCTs. This meta-analysis verified that the effectiveness of atropine in controlling myopia progression was closely related to the dose. 0.05% atropine might be the optimal dose which could slow the myopia progression and had the least adverse effects and rebound after discontinuation. Although only one study in our meta-analysis confirmed the effectiveness of 0.05% atropine, the study was of good quality after the Methodological Evaluation and was currently the largest placebo-controlled RCT to comprehensively evaluate its safety and effectiveness. In our study, the same conclusion is also got. Therefore, the study is sufficient to indicate that 0.05% may be the best dose of atropine according to all the doses of atropine in this meta-analysis. If atropine can be widely used in clinical prevention and controlling myopia, it will help prevent high myopia and related complications.

Kinoshita reported that the combined application of 0.01% atropine eye drops and orthokeratology can significantly slow the axis elongation compared to the use of orthokeratology alone [28]. A retrospective study also reported similar results [29]. But the elongation of the eye axis could not predict the progression of myopia accurately. Therefore, the effectiveness of the combined application of atropine and orthokeratology needed to be further studied.

There were several limitations. First, although this meta-analysis had established strict inclusion and exclusion criteria, the heterogeneity was still high after using the subgroup analysis. However, through the sensitivity analysis, the results of this meta-analysis were stable and consistent. Secondly, there were no studies involving 0.01% atropine in this study. And some of the included studies did not report adverse reactions, and few studies reported the progression of myopia after atropine was discontinued. The further determination and validation of the optimal dose required additional research.

## Conclusions

The effectiveness of atropine in controlling the progression of myopia was closely related with dose. A 0.05% atropine was likely to be the optimal dose.

## Supplementary Information

**Additional file 1.**

## Data Availability

All data are available under request. Congling Zhao should be contacted if someone wants to request the data.

## Abbreviations

RCTs: Randomized controlled trials
CI: Confidence interval

## References

[1] Morgan IG, French AN, Ashby RS, Guo X, Ding X, He M, Rose KA (2018). The epidemics of myopia: Aetiology and prevention. Prog Retin Eye Res.

[2] Dong L, Kang YK, Li Y, Wei WB, Jonas JB (2020). PREVALENCE AND TIME TRENDS OF MYOPIA IN CHILDREN AND ADOLESCENTS IN CHINA: a systemic review and meta-analysis. Retina.

[3] Grzybowski A, Kanclerz P, Tsubota K, Lanca C, Saw SM (2020). A review on the epidemiology of myopia in school children worldwide. BMC Ophthalmol.

[4] Chen M, Wu A, Zhang L, Wang W, Chen X, Yu X, Wang K (2018). The increasing prevalence of myopia and high myopia among high school students in Fenghua city, eastern China: a 15-year population-based survey. BMC Ophthalmol.

[5] Spillmann L (2020). Stopping the rise of myopia in Asia. Graefe's Arch Clin Exp Ophthalmol.

[6] Gifford KL, Gifford P, Hendicott PL, Schmid KL (2020). Stability of peripheral refraction changes in orthokeratology for myopia. Cont Lens Anterior Eye.

[7] Guan M, Zhao W, Geng Y, Zhang Y, Ma J, Chen Z, Peng M, Li Y (2020). Changes in axial length after orthokeratology lens treatment for myopia: a meta-analysis. Int Ophthalmol.

[8] Upadhyay A, Beuerman RW (2020). Biological mechanisms of atropine control of myopia. Eye Contact Lens.

[9] Schittkowski MP, Sturm V (2018). Atropine for the prevention of progression in myopia - data, side effects, practical guidelines. Klinische Monatsblatter fur Augenheilkunde.

[10] Chua WH, Balakrishnan V, Chan YH, Tong L, Ling Y, Quah BL, Tan D (2006). Atropine for the treatment of childhood myopia. Ophthalmology.

[11] Hsiao CK, Tsai MY, Chen HM (2005). Inference of nested variance components in a longitudinal myopia intervention trial. Stat Med.

[12] Kumaran A, Htoon HM, Tan D, Chia A (2015). Analysis of changes in refraction and biometry of atropine- and placebo-treated eyes. Invest Ophthalmol Vis Sci.

[13] Polling JR, Kok RG, Tideman JW, Meskat B, Klaver CC (2016). Effectiveness study of atropine for progressive myopia in Europeans. Eye.

[14] Shih YF, Hsiao CK, Chen CJ, Chang CW, Hung PT, Lin LL (2001). An intervention trial on efficacy of atropine and multi-focal glasses in controlling myopic progression. Acta Ophthalmol Scand.

[15] Shih YF, Chen CH, Chou AC, Ho TC, Lin LL, Hung PT (1999). Effects of different concentrations of atropine on controlling myopia in myopic children. J Ocul Pharmacol Ther.

[16] Wang YR, Bian HL, Wang Q (2017). Atropine 0.5% eyedrops for the treatment of children with low myopia: A randomized controlled trial. Medicine.

[17] Yam JC, Jiang Y, Tang SM, Law AKP, Chan JJ, Wong E, Ko ST, Young AL, Tham CC, Chen LJ (2019). Low-concentration atropine for myopia progression (LAMP) study: a randomized, double-blinded, placebo-controlled trial of 0.05, 0.025, and 0.01% atropine eye drops in myopia control. Ophthalmology.

[18] Yen MY, Liu JH, Kao SC, Shiao CH (1989). Comparison of the effect of atropine and cyclopentolate on myopia. Ann Ophthalmol.

[19] Yi S, Huang Y, Yu SZ, Chen XJ, Yi H, Zeng XL (2015). Therapeutic effect of atropine 1% in children with low myopia. J AAPOS.

[20] Moon JS, Shin SY (2018). The diluted atropine for inhibition of myopia progression in Korean children. Int J Ophthalmol.

[21] Lee JJ, Fang PC, Yang IH, Chen CH, Lin PW, Lin SA, Kuo HK, Wu PC (2006). Prevention of myopia progression with 0.05% atropine solution. J Ocul Pharmacol Ther.

[22] Song YY, Wang H, Wang BS, Qi H, Rong ZX, Chen HZ (2011). Atropine in ameliorating the progression of myopia in children with mild to moderate myopia: a meta-analysis of controlled clinical trials. J Ocul Pharmacol Ther.

[23] Gong Q, Janowski M, Luo M, Wei H, Chen B, Yang G, Liu L (2017). Efficacy and adverse effects of atropine in childhood myopia: a meta-analysis. JAMA Ophthalmol.

[24] Larkin GL, Tahir A, Epley KD, Beauchamp CL, Tong JT, Clark RA (2019). Atropine 0.01% eye drops for myopia control in American children: a multiethnic sample across three US sites. Ophthalmol Ther.

[25] Zhao Y, Feng K, Liu RB, Pan JH, Zhang LL, Xu ZP, Lu XJ (2019). Atropine 0.01% eye drops slow myopia progression: a systematic review and meta-analysis. Int J Ophthalmol.

[26] Tong L, Huang XL, Koh AL, Zhang X, Tan DT, Chua WH (2009). Atropine for the treatment of childhood myopia: effect on myopia progression after cessation of atropine. Ophthalmology.

[27] Chia A, Chua WH, Wen L, Fong A, Goon YY, Tan D (2014). Atropine for the treatment of childhood myopia: changes after stopping atropine 0.01, 0.1 and 0.5%. Am J Ophthalmol.

[28] Kinoshita N, Konno Y, Hamada N, Kanda Y, Shimmura-Tomita M, Kakehashi A (2018). Additive effects of orthokeratology and atropine 0.01% ophthalmic solution in slowing axial elongation in children with myopia: first year results. Jpn J Ophthalmol.

[29] Chen Z, Huang S, Zhou J, Xiaomei Q, Zhou X, Xue F (2019). Adjunctive effect of orthokeratology and low dose atropine on axial elongation in fast-progressing myopic children-a preliminary retrospective study. Cont Lens Anterior Eye.

